# Reversine Induced Multinucleated Cells, Cell Apoptosis and Autophagy in Human Non-Small Cell Lung Cancer Cells

**DOI:** 10.1371/journal.pone.0158587

**Published:** 2016-07-06

**Authors:** Yin-Che Lu, Ying-Ray Lee, Ji-Der Liao, Ching-Yen Lin, Yih-Yuan Chen, Ping-Tzu Chen, Ya-Shih Tseng

**Affiliations:** 1 Department of Health and Nutrition, Chia Nan University of Pharmacy and Science, Tainan, Taiwan; 2 Division of Hematology-Oncology, Ditmanson Medical Foundation Chia-Yi Christian Hospital, Chiayi, Taiwan; 3 Department of Medical Research, Ditmanson Medical Foundation Chia-Yi Christian Hospital, Chiayi, Taiwan; 4 Department of Medical Laboratory Science and Biotechnology, College of Medicine and Life Science, Chung Hwa University of Medical Technology, Tainan, Taiwan; 5 Department of Biochemical Science and Technology, National Chiayi University, Chiayi, Taiwan; 6 Department of Internal Medicine, Ditmanson Medical Foundation Chia-Yi Christian Hospital, Chiayi, Taiwan; Virginia Commonwealth University, UNITED STATES

## Abstract

Reversine, an A3 adenosine receptor antagonist, has been shown to induce differentiated myogenic-lineage committed cells to become multipotent mesenchymal progenitor cells. We and others have reported that reversine has an effect on human tumor suppression. This study revealed anti-tumor effects of reversine on proliferation, apoptosis and autophagy induction in human non-small cell lung cancer cells. Treatment of these cells with reversine suppressed cell growth in a time- and dosage-dependent manner. Moreover, polyploidy occurred after reversine treatment. In addition, caspase-dependent apoptosis and activation of autophagy by reversine in a dosage-dependent manner were also observed. We demonstrated in this study that reversine contributes to growth inhibition, apoptosis and autophagy induction in human lung cancer cells. Therefore, reversine used as a potential therapeutic agent for human lung cancer is worthy of further investigation.

## Introduction

Lung cancer is the most common cause of cancer-related death worldwide, and has been classified as small cell lung cancer (SCLC) and non-small cell lung cancer (NSCLC). Of the two, NSCLC accounts for 75–80% of primary lung cancer patients. However, the biologic and clinical features of SCLC are considered different with other lung cancers. SCLC exhibits aggressive behavior, and is the most malignancy of various lung cancers [1–3]. Surgery is the most effective therapeutic modality to achieve a cure, but the postoperative prognosis is poor. In addition, most patients present in advanced stages, and for them, chemotherapy with or without radiotherapy is recommended. Recrudesce and disease progression may appear quickly in NSCLC and SCLC patients, and the prognosis is poor [2, 3]. Therefore, a novel and effective treatment modality is urgently needed for both SCLC and NSCLC.

Reversine, a small molecule, was originally identified to induce dedifferentiation of murine myoblasts into multipotent progenitor cells [4]. Later, the role of reversine in anti-tumor activities was promoted in human myeloid leukemia, multiple myeloma, cervical carcinoma, thyroid cancer, breast cancer, oral squamous cell carcinoma and prostate cancer [5–11]. Reversine has been shown to suppress the proliferation of multiple human cancer cells, through actions such as cell cycle arrest, apoptosis and autophagy induction [5–11]. Moreover, reversine has been reported to be a potent Aurora kinases (Aur) inhibitor, and inhibits acute myeloid leukemia growth as well as VX-680, but is less toxic [5]. In addition, reversine is an ATP analogue and is speculated to be an inhibitor for various enzymatic activities, including Aurora kinase [6]. Reversine has been shown to inhibit cancer cells through the cell cycle regulator proteins Aurora kinase-A (Aur-A) and -B (Aur-B), JAK2 and SRC [5, 6]. Therefore, reversine could be a novel anticancer agent for multiple cancers. However, the antitumor behavior of reversine has not yet been clearly elucidated in human lung cancers.

In the present study, we demonstrated that reversine can suppress cell growth and inhibit the colony formation of human NSCLC cells. Moreover, Aur suppression and polyploidy cells were found with reversine treatment. Apoptosis and autophagy also occurred after reversine treatment. Therefore, our data suggest that reversine can be used as an anticancer agent in human NSCLC.

## Materials and Methods

### Lung cancer cell lines and cell culture

Two human lung cancer cells lines, A549 and H1299, were chosen in the study. A549 cells are p53 normal, and H1299 cells are p53 null. The H1299 and A549 cells were maintained in RPMI1640 medium (Gibco BRL, Grand Island, NY) and DMEM (Gibco) with 10% FBS (Gibco), respectively. The cells were incubated at 37°C in 5% CO2.

### Cell proliferation assay

Reversine was purchased from Cayman Chemical (Ann Arbor, Michigan, USA). A549 and H1299 cells (5 × 10^3^/well) were plated into 96-well tissue culture plates and grown with the above mentioned medium. Cells were treated with medium only (containing 0.01% DMSO as the negative control) or medium containing reversine at 0.5, 1, 5, 10 and 20 μM. After incubation for 24, 48, and 72 hours, the cell viability were determined by MTT assay. Three replicates were performed and analyzed.

### Colony formation analysis

To determine the long-term effects of transient drug exposure in cells, cells were seeded (300 cells/well) in a 6-well culture dish, and were incubated with or without reversine for 72 hours. After the cells had been rinsed with fresh medium, they were allowed to grow for 14 days to form colonies, and were stained with crystal violet. Three replicates were performed and analyzed.

### Measurement of multinucleated cells

Cells were treated with or without reversine for 72 hours. The nuclei were stained with DAPI (Sigma, St. Louis, MO) for 20 min at room temperature (RT). The morphology of multi-nuclei (two or more nuclei in one cell) was confirmed under a fluorescent microscope and compared with the observations obtained under the light field.

### Cell cycle analysis

Cells were incubated with DMSO or reversine (5mM) for 24, 48 and 72 hours, and then were harvested and fixed with 70% ethanol overnight. Cells were then labeled with PI (Sigma) and incubated at RT in the dark for 30 min. DNA content was analyzed using Millipore (Becton–Dickinson, San Diego, CA).

### Apoptosis analysis

Annexin-V staining (Sigma) of cells treated with DMSO or reversine was performed to detect apoptotic cells. Cells after staining were washed with PBS, and then the cell pellets were resuspended in staining solution (Annexin-V-FITC and PI) and incubated for 15 min at room temperature in darkness. Annexin-V or PI fluorescent intensities were analyzed by FACScan (Becton–Dickinson, San Diego, CA), and 10,000 cells were evaluated in each sample.

### Western Blotting

To illustrate the occurrence of apoptosis and autophagy in reversine-treated cells, the activation of caspase-3, and PARP expression were determined by Western blot (all of the antibodies were purchased from Cell Signaling Technology, Inc. Danvers, MA). The induction of autophagy by reversine was evaluated by the expression of LC3-II (antibody was purchased from Medical & Biological Laboratories Co., Nagoya, Japan) using Western blot. The expression of β-actin (antibody was purchased from Cell Signaling Technology, Inc.) was also determined as an internal control.

### Statistical analysis

Data are presented as mean ± standard deviation for the indicated number of separate experiments. Statistical evaluation was carried out by Student’s *t*-test.

## Results

### Reversine inhibits cell growth and colony formation of human lung cancer cells

**Fig 1:**
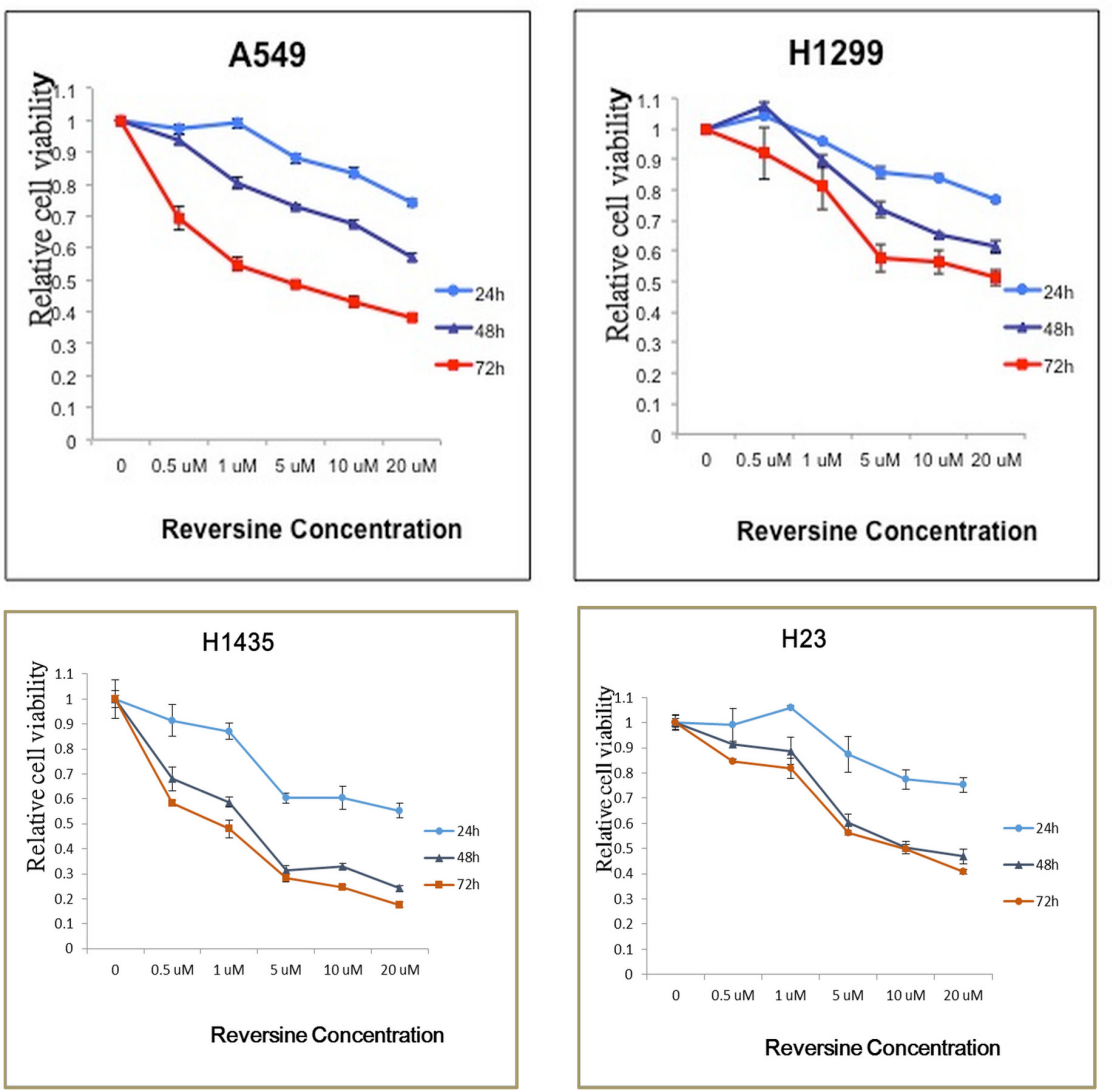
Reversine inhibited the growth of human NSCLC cells. Cells were incubated with reversine for 24, 48 and 72 h, and the cellular viability of four cell lines including A549, H1299, H1435 and H23 cells was analyzed by MTT assay. All of the data were expressed as mean ± SEM of three independent experiments.

**Fig 2:**
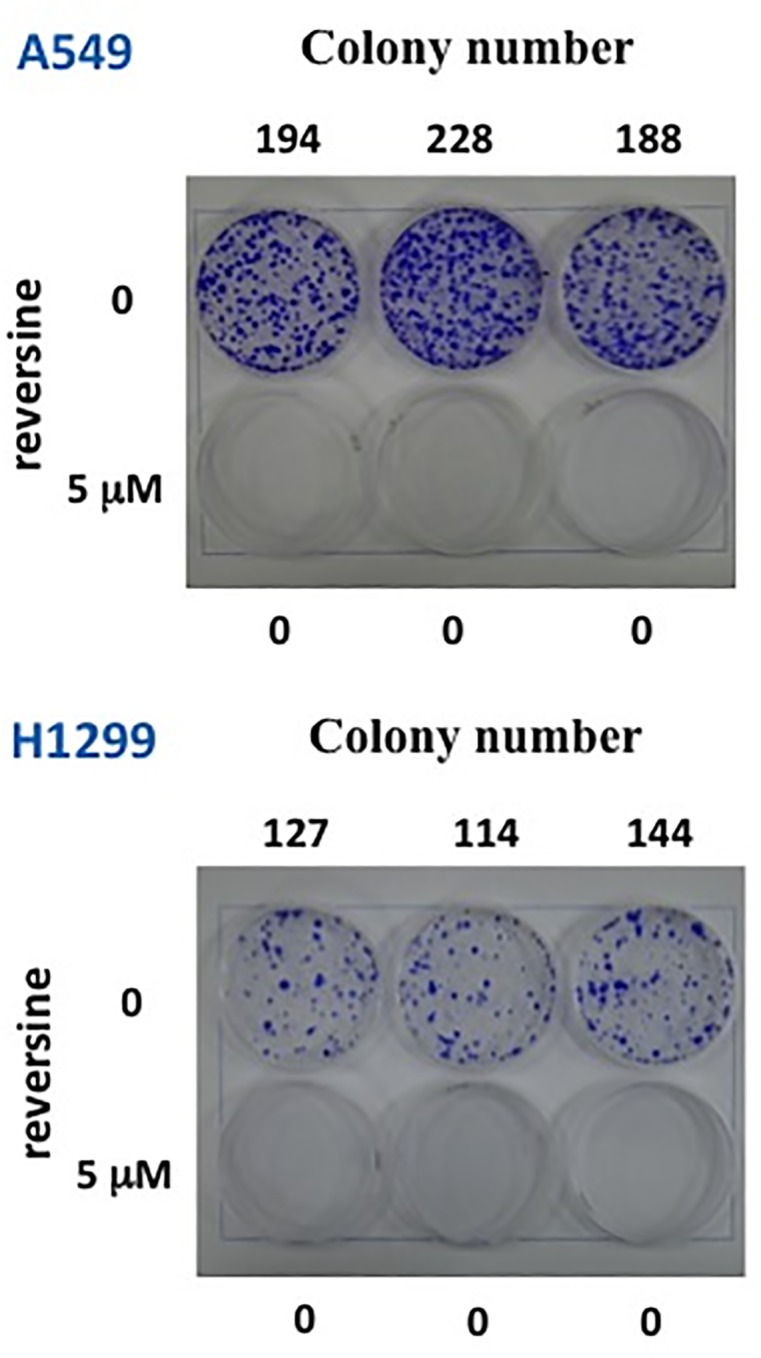
Reversine suppressed colony formation in human NSCLC cells. Cells were exposed to reversine or not, and the colony formation of these cells was determined by crystal violet staining.

Two human NSCLC cell lines, A549 and H1299, were used to evaluate the growth inhibition effect after reversine treatment. DMSO was used as a negative control. Cell viability of all cells treatment with reversine was decreased significantly in a time- and dosage-dependent manner. The IC_50_ of reversine in A549, H1299, H1435 and H23 cells was 4, 20, 0.9 and 9.7 μM after 72 hours of treatment, respectively (Fig 1). Notably, A549 and H1435 cells were more sensitive to reversine as compared with other tested cell lines. To clarify whether reversine could suppress tumor formation *in vitro*, colony formation analysis was also used to evaluate the anti-tumor benefit of reversine in human NSCLCs. Reversine could inhibit the numbers of colony formations in the two cells (Fig 2). These data demonstrated that reversine could suppress cell growth and tumor formation *in vitro* in human NSCLC cells.

### Reversine induces multinucleated cells in human NSCLC cells

**Fig 3:**
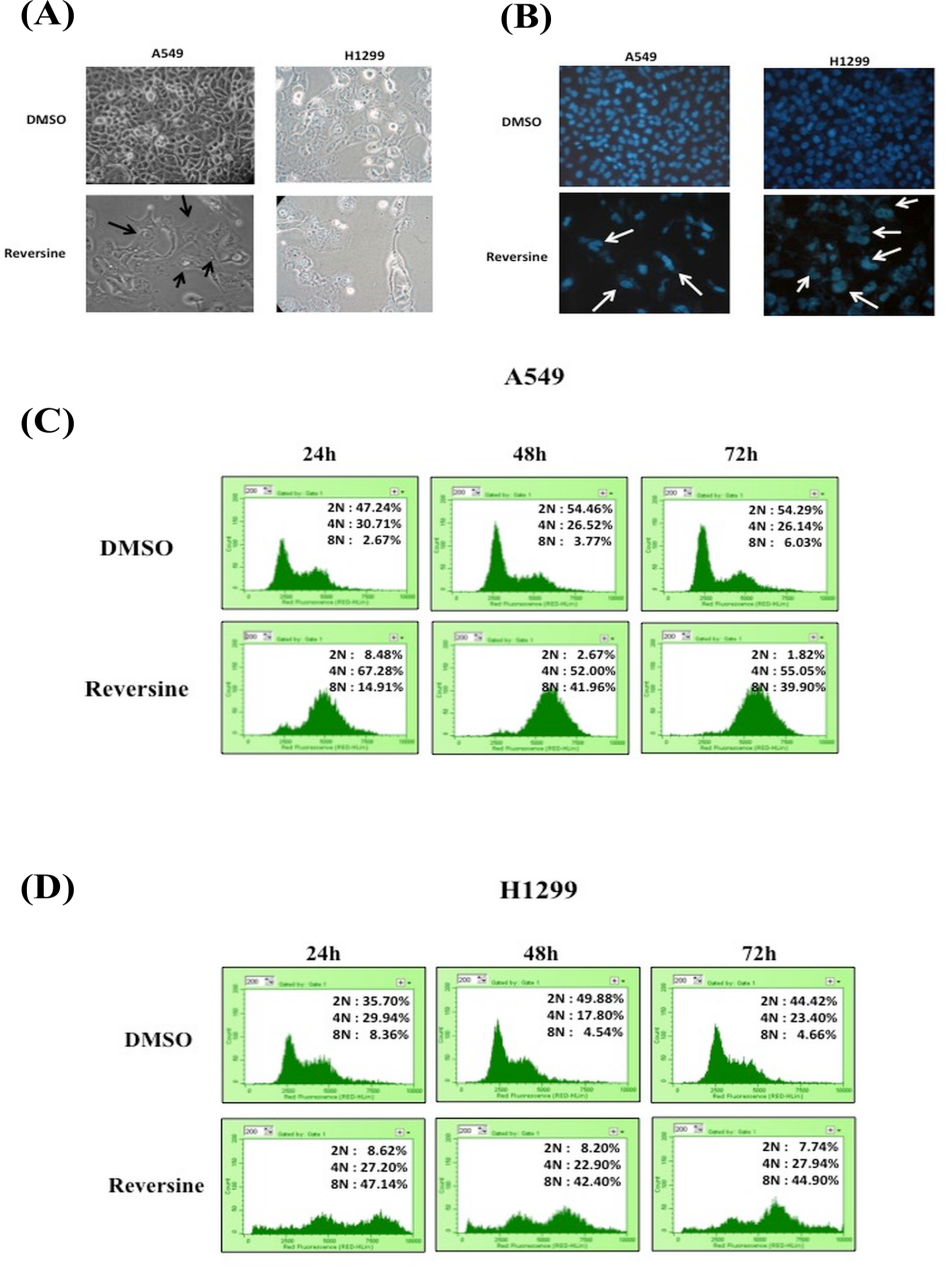
Reversine induced multinuclear cell formation in human NSCLC cells. (A) Cells were incubated with reversine for 72 h, and the cellular morphology was determined under microscopy. (B) The cellular nuclear was stained and determined under fluorescence microscopy. The multinuclear cells are indicated by an arrow. (C, D) The DNA content of the cells was determined by flow cytometry with propidium iodide labeling. The percentage of 2N, 4N and 8N distributions in reversine-treated cells was measured with WMDI 2.9 software. Two independent experiments were confirmed and one of them was shown. DMSO was used as a negative control.

**Fig 4:**
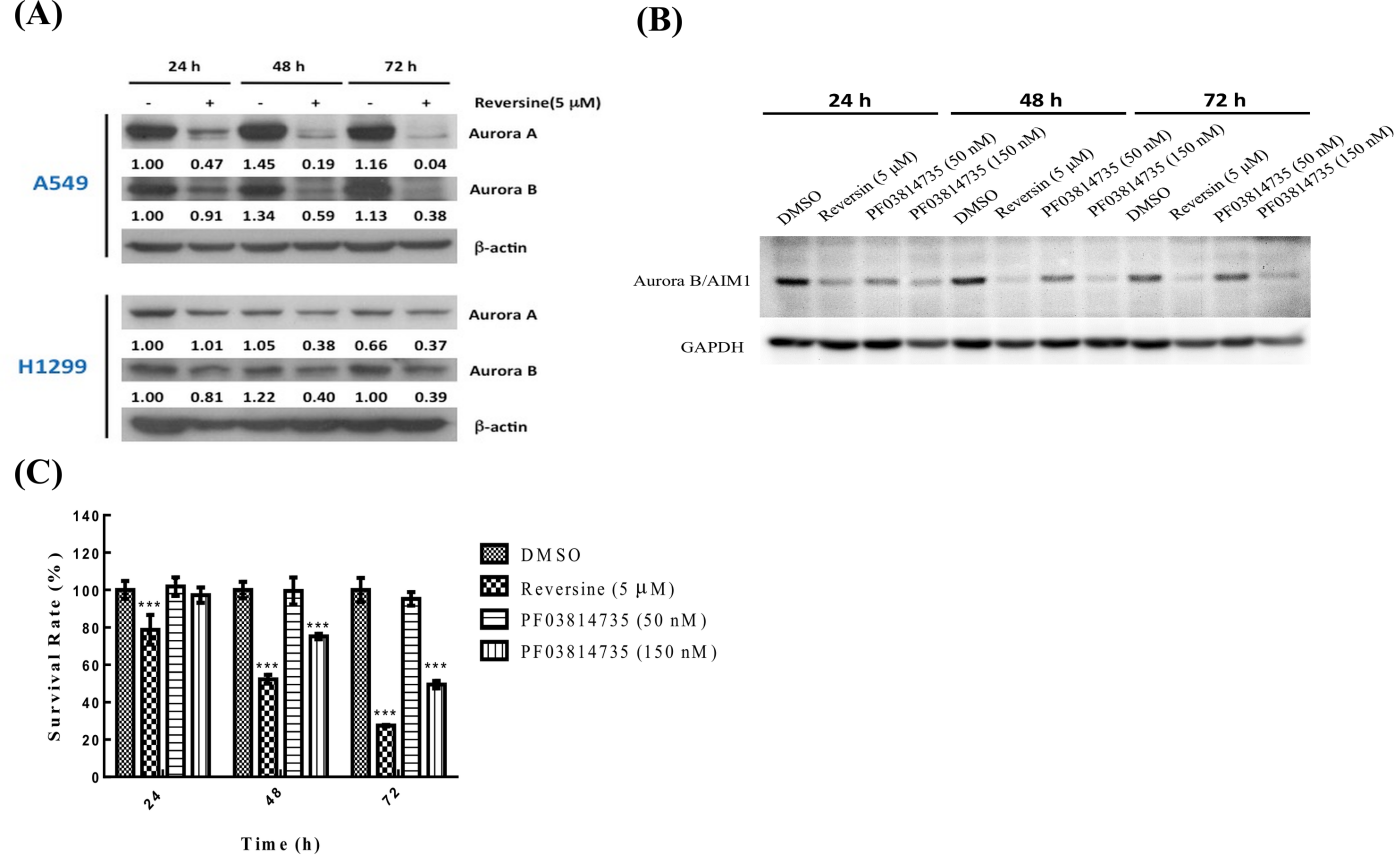
Aur expressions in human NSCLC cells under reversine treatment. (A) Cells were incubated with or without reversine, and the expressions of Aur-A and Aur-B were determined by Western blotting. β-actin was used as a loading control. (B) Cells were incubated with reversine or inhibitor of Aur-A/B, PF03814735. Following the expression of Aur-B/AIM was determined. GAPDH was used as a loading control. (C) The cells were treated with reversine or PF03814735, respectively. At selected time points, the cell viability was determined by MTT assay.

The formation of giant multinucleated cells is a phenotype of mitotic catastrophe induced by ionizing radiation or certain anticancer drugs [12]. In addition, the induction of polyploidy in human prostate and breast cancer cells by reversine has been reported [5, 10, 11]. Therefore, we investigated whether reversine induced multinucleation in these two cell lines. Under microscopy, the cells containing an enlarged phenotype could be observed after reversine treatment (Fig 3A). This phenomenon was confirmed by nucleo-staining and the multinucleated cells were observed in the reversine-treated groups in both cell lines (Fig 3B). In addition, a method for determining the cell cycle by flow cytometry was also used to evaluate the multinucleated cells after reversine treatment. Polyploid cells were detected in reversine-treated A549 and H1299 cells (Fig 3C and 3D). This data suggested that reversine-treated cells could exit mitosis without cell division. Reversine has been demonstrated to be an inhibitor of Aur [5], and it has been reported that Aur regulate mitosis [13], so we further investigated whether reversine could influence Aur-A and B in human NSCLC cells. We found that reversine could reduce the expression level of Aur-A and -B in human A549 and H1299 cells (Fig 4A). Moreover, using PF03814735, an inhibitor of Aur-A and -B, showing suppression of the expression level of Aur-B (Fig 4B). In addition, A549 cells treated with PF03814735 reducing cell viability as well as reversine treatment (Fig 4C), suggesting inhibition of Aur-A or–B could suppress cellular proliferation of human NSCLC cells. Altogether, reversine indeed induced a mitotic catastrophe phenomenon in human NSCLC cell lines and the protein levels of Aur-A/B were involved.

### Reversine induces caspase-dependent apoptosis in human NSCLC cells

**Fig 5:**
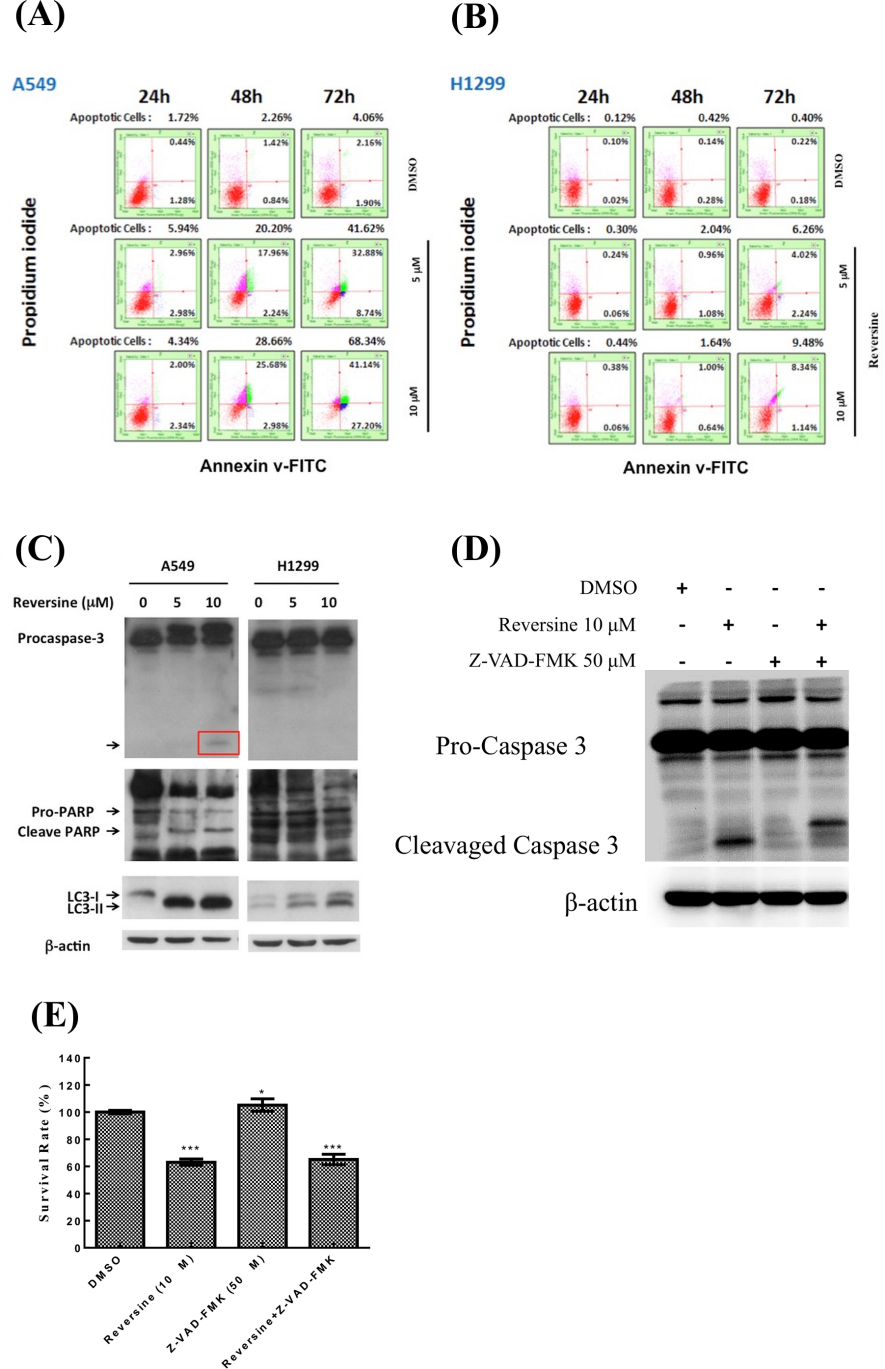
Detection of reversine-mediated apoptosis in human NSCLC cells. Cells were treated with DMSO or reversine, and the apoptotic cells of (A) A549, and (B) H1299 cells were assessed by flow cytometry analysis with PI/Annexin-V double staining. (C) Cells were incubated with DMSO or reversine for 72 h, and the expressions of the cleavage form of caspase-3, PARP and LCII were determined by Western blotting. β—actin was used as a loading control. Two independent experiments were confirmed, and one of them was shown. (D) The cells were treated with reversine or Z-VAD-FMK, respectively. Following the expression of caspase-3 was determined. β-actin was used as a loading control. (E) The cells were treated with reversine, PF03814735 or reversine/PF03814735 respectively. At selected time point, the cell viability was determined by MTT assay.

The induction of apoptosis by reversine has been reported by us and other groups [7–9, 11, 14, 15]. In this study, flow cytometry analysis was used to confirm whether apoptosis occurred in human NSCLC cells under reversine treatment. The cells were incubated with DMSO or reversine. Reversine induced apoptosis in human NSCLC cells in a time- and dosage-dependent manner (Fig 5A and 5B). We found that A549 cells were more susceptible to reversine-mediated apoptosis than H1299 cells. To evaluate the occurrence of apoptosis and the involvement of caspase in reversine-treated human NSCLC cells, Western blotting was used to detect the activation of PARP and caspase-3. Cleaved caspase-3 was observed in reversine-treated A549 cells, in a dosage-dependent manner (Fig 5C). Moreover, apoptosis induced by reversine and evaluated by observation of cleaved PARP in the A549 cells consistently showed cleaved caspase 3 (Fig 5C), and only cleavage PARP was observed in H1299 cells. These data revealed that A549 cells were more susceptible to reversine-induced caspase-dependent apoptosis than H1299 cells that contained a null mutation of p53. Moreover, further study illuminated that inhibition of caspase-dependent apoptosis with pan-caspase inhibitor (Z-VAD-FMK) can’t reverse reversine-mediated cell death (Fig 5D and 5E), suggesting that apoptosis may not be the only reasons for reversine-mediated cell death.

### Reversine induces autophagosome formation in human NSCLC cells

**Fig 6:**
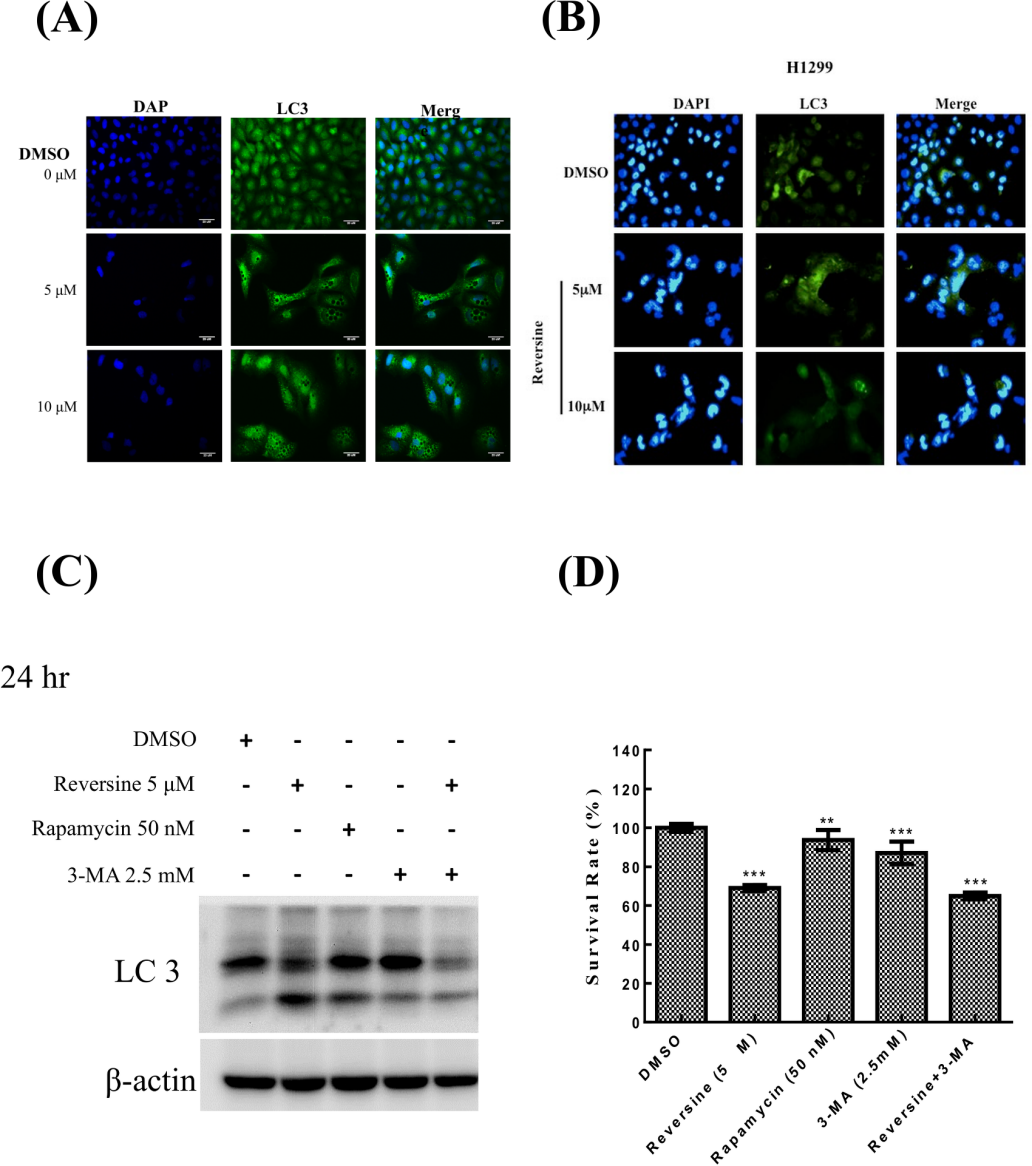
Autophagy induced by reversine in human NSCLC cells. Cells were treated with reversine for 72 h, and following the formation of autophagosome were observed by florescence-microscopy using methods as described in materials and methods. (A) A549 cells; (B) H1299 cells. (C) In order to clarify that reversine induces autophagosome formation, an inducer (rapamycin) and an inhibitor (3-MA) of autophagy were selected and tested. The cells were treated with reversine, rapamycin and 3-MA, respectively. Following the expression of LC-3 was determined. β-actin was used as a loading control. (D) The cells were treated with reversine, rapamycin, 3-MA and reversine/3-MA, respectively. At selected time point, the cell viability was determined by MTT assay.

The induction of autophagy by reversine in human follicular thyroid cancer and oral squamous cell carcinoma has been reported [8, 9]. In the present study, we also investigated whether autophagosome was elevated during reversine treatment in human NSCLC cells. To examine this, cells were treated with DMSO or reversine and the expression of LC3-II, a marker of autophagosome, was determined by Western blotting. The expression of LC3-II in A549 and H1299 cells after reversine treatment will be elevated in a dosage-dependent manner (Fig 5C). In addition, formation of autophagosome was also determined in A549 and H1299 cells (Fig 6A and 6B). Our results revealed that the expression of LC3-II was significantly higher in A549 cells than in H1299 cells (Fig 5C). To confirm that autophagy was indeed happen under reversine treatment, an inhibitor of autophagy was used to suppress this phenomenon. Fig 6C showed that reversine can elevate LC3-II as well as rapamycin treatment, and 3-MA can reduce reversine mediated LC3-II overexpression. These data illuminated that reversine can induce autophagy in human NSCLC cells. In addition, suppression of autophagy with 3-MA treatment can’t reverse reversine-mediated cell death, suggesting that reversine-mediated autophagy may nlt be the only reasons for reversine-mediated cell death (Fig 6C and 6D). Altogether, our investigations suggested that A549 that contaned p53 was more susceptible to reversine-induced autophagosome formation than H1299 that contained mutant p53. The involvement of p53 in reversine-induced apoptosis or autophagosome formation will be further investigated.

## Discussion

In the present study, we demonstrated an antitumor ability of reversine in four human NSCLC cell lines. Of the four cell lines, A549 and H1435 cells were more susceptible to growth inhibition, as well as apoptosis and autophagosome formation, by reversine. The anti-tumor behavior of reversine has also been demonstrated in multiple cancers including of melanoma, oral cancer, breast cancer, thyroid cancer, colon cancer, cervical cancer, etc. [5, 7–9, 11, 14, 15], and our findings in this study are similar with these reports. The IC50 of reversine in the reported cancer cells are 1 to 20 μM. Among them, leukemia cancer cells are more sensitive for reversine (IC50 = 0.1 μM) treatment [5].

Moreover, reversine also induced a multinucleated phenomenon of the cells (Fig 3). These findings are consistent with the observations in MCF-7, MDA-MB-231, HeLa, CWR22Rv1, DU-145, and PC-3 cells after reversine treatment [10, 11, 16]. Aur, composed of Aur-A, Aur-B, and Aur-C, belong to the serine/theonine kinase family. Aur are the key regulators of mitosis [13]. Aur-A and/or Aur-B overexpression has been reported in multiple types of tumors, including NSCLC, thyroid, breast, colon, pancreatic, ovarian, gastric, prostate, and glioblastoma, and contributed to tumor grade and prognosis [17–28]. Identification and development of Aur inhibitors has been the subject of treatment of human cancers in previous studies [29–31]. Reversine has been reported to be an Aur inhibitor [5]. Here, we demonstrated that reversine mediated multinuclear formation in human NSCLC cells (Fig 3). As such, whether reversine can suppress human NSCLC cell growth through inhibition of Aur and/or other factors should be further investigated.

In addition, reversine has been shown to inhibit multiple enzymes, including JAK2, Src, and Akt, which are reported to be involved in the signaling pathways of cell growth or metastasis [4, 6]. Activation or overexpression of JAK2, Src and Akt pathways contributes to tumor growth, angiogenesis, metastasis, and/or invasion of human NSCLC cells [32–39]. Overexpression of JAK2, Src and Akt pathways can elevate the resistance of tumors to chemotherapy and radiotherapy [40–44]. Inhibition of the JAK2/STAT, Src and/or Akt pathway showed antitumor activity through growth inhibition, apoptosis induction and/or metastasis/invasion inhibition [38, 40, 45]. Therefore, whether reversine can suppress human NSCLC tumor growth and induce cancer death through the inhibition of these pathways should be further investigated. The combination of reversine with current clinical therapeutic agents as an antitumor cocktail therapy also needs to be studied.

The induction of apoptosis by reversine has been reported in several human tumors [7, 9, 11, 14, 15]. However, this is the first time to observe the induction of apoptosis by reversine in human NSCLS cells. Moreover, caspase-dependent apoptosis mediated by reversine was also illustrated in this study (Fig 5C), as well as in other reports [9, 11, 14, 15]. We found that A549 and H1435 were more susceptible to reversine treatment, which induced a high level of apoptosis and autophagosome formation. Notably, Jemaa *et al*. have demonstrated that reversine induces cellular apoptosis, particularly in p53-deficient cancer cells [14]. However, our study showed that A549 harboring normal p53 was more sensitive to reversine than H1299 and H23 cells with a null p53 mutation. Therefore, whether others, besides p53, are involved in reversine-induced apoptosis in human NSCLC cells needs further investigation.

The induction of autophagy by reversine has been reported in other studies [8, 9]. In this study, we demonstrated that reversine could induce autophagy in human NSCLC cells. Overexpression of Aur-A has been demonstrated to suppress autophagy; however, inhibition of Aur-A by siRNA or VX-680 showed an induction of autophagy in human breast cancer cells [46]. Another report also confirmed this finding in human NSCLC cells [47]. Therefore, whether reversine-mediated autophagy induction is through Aur inhibition also needs further study.

## Conclusions

In the present study, we reported the anti-tumor activities *in vitro* of reversine on human NSCLC cells including multinuclear cell formation, apoptosis and/or autophagy. A549 cells are more susceptible for the treatment of reversine than H1299 cells. We also demonstrated that caspase-dependent pathway is involved in reversine mediated apoptosis in human NSCLC cells. Altogether, reversine is a potential therapeutic agent against human NSCLCs and is worthy of further clinical investigation.
